# X-Ray Diffraction of Collagen-Structured Water Molecules for Cancer Detection

**DOI:** 10.3390/molecules31040650

**Published:** 2026-02-13

**Authors:** Sasha Murokh, Alexander Alekseev, Viacheslav Kubytskyi, Viacheslav Shcherbakov, Oleksii Avdieiev, Sergey A. Denisov, Ashkan Ajeer, Lois Adams, Charlene Greenwood, Keith Rogers, Lev Mourokh, Pavel Lazarev

**Affiliations:** 1 Matur UK Ltd., 5 New Street Square, London EC4A 3TW, UKplazarev@matur.co.uk (P.L.); 2 Department of Computer Science, Rensselaer Polytechnic Institute, Troy, NY 12180, USA; 3 Department of Physics and Technology, Karaganda Buketov University, Karaganda 100028, Kazakhstan; 4 Laboratoire de Physique des 2 Infinis Irène Joliot-Curie, UMR9012, CNRS, Université Paris-Saclay, Bât. 209, 91405 Orsay, France; 5 Institut de Chimie Physique, UMR8000, CNRS, Université Paris-Saclay, Bât. 349, 91405 Orsay, France; 6 School of Chemical and Physical Sciences, Keele University, Keele ST5 5BG, UKc.e.greenwood@keele.ac.uk (C.G.); 7 EosDx, Inc., 15211 Vanowen Street, Suite 209, Los Angeles, CA 91405, USA; k.d.rogers@cranfield.ac.uk; 8 Shrivenham Campus, Cranfield University, Swindon SN6 8LA, UK; 9 Physics Department, Queens College, City University of New York, 65-30 Kissena Blvd, Flushing, NY 11367, USA

**Keywords:** cancer detection, X-ray scattering, structural biomarkers, collagen, fibroadenoma, water molecules

## Abstract

Structural biomarkers determined by X-ray scattering of the tissues can complement conventional histopathology and facilitate a fast triage procedure of cancer biopsy samples. It has been shown previously that lipid reflexes can distinguish cancerous from benign samples, except for fibroadenomas. In the present study, we demonstrate that fibroadenoma samples can be recognized using X-ray scattering of collagen. Moreover, we show that modifications in collagen structure are manifested in the water reflexes. Examination of diffraction patterns from water using two-dimensional Fourier transformation and machine learning yields excellent classification metrics in both synchrotron images and laboratory diffractometer data.

## 1. Introduction

Breast cancer is the most commonly diagnosed cancer among women worldwide. According to the predictions of the Lancet’s Breast Cancer Commission [1], by 2040, there will be more than 3 million new cases of breast cancer per year. Of women in the US, 13.1% will be diagnosed with invasive breast cancer, and 2.3% will die from the disease [2]. In 2024, an estimated 310,720 new cases of invasive breast cancers and 56,500 new cases of ductal carcinoma in situ will be diagnosed among women and 2790 among men in the US [2]. The death rate has decreased recently [3], mainly due to advances in early diagnostics [4,5]. Correspondingly, avoiding any delays in cancer detection is paramount for a patient. Both cancerous and benign lesions require histopathological examination; biopsies with a benign diagnosis being eight times more likely than those with a malignant one. Currently, there is no opportunity to ‘fast-track’ potential malignant cases. Thus, there is a real need to adopt a different methodology for tissue diagnostics that can effectively triage samples at an early stage, allowing identification of those with potentially life-threatening conditions and, ideally, offering early reassurance to those without significant disease.

Such an approach for breast cancer detection can be implemented by exploiting the structural biomarkers obtained from X-ray scattering of human breast tissues. In particular, wide-angle X-ray scattering (WAXS), with momentum transfer (*q*) approximately between 3 and 40 nm^−1^, provides information on variations in lipid and aqueous components [6,7,8,9,10,11,12,13,14]. It was shown [9,14] that in cancerous tissues, the intensity of a maximum at ~*q* = 14 nm^−1^ (corresponding to inter-fatty-acid molecular distances) is reduced. Concurrently, at ~*q* = 20 nm^−1^, the intensity of a maximum (associated with the oxygen–oxygen distance in the tetrahedral structure of water) increases. This effect can serve as a structural biomarker for breast cancer detection, although the data have been mostly derived from synchrotron experiments with limited sample numbers. Recently, we have employed bespoke *laboratory* diffractometers for the same purposes and observed the same maxima variations [15,16,17] across a large number of samples in both the US [15] and the UK [16,17]. Specifically, using machine learning for binary benign/cancer differentiation, we achieved 95% sensitivity and 100% specificity [17]. However, in this analysis, fibroadenoma was excluded from the benign dataset because its lipid signal is very similar to that of cancer.

The momentum transfer range, 0.1 < *q* < 5 nm^−1^, corresponding to small-angle scattering (SAXS), provides information about possible alterations in the collagen fibril repeat distances and coherence [18,19,20,21,22], the amorphous scattering profile [6,20], and triglyceride packing (*q* = 1.5 nm^−1^) [14,23]. In this paper, we demonstrate, for the first time, the feasibility of separating fibroadenoma samples from cancerous samples using collagen features. In the SAXS images obtained at the Diamond Light Source, we removed the triglyceride peak and evaluated the reflexes at smaller angles. The signal from collagen fibrils is anisotropic; thus, we used a recently developed approach based on two-dimensional Fourier coefficients, principal component analysis, and a logistic regression classifier [17,24,25]. We demonstrated that the patterns from cancerous and fibroadenoma samples are well separated in the space of principal components, with an area under the receiver operating characteristic (ROC) curve (AUC) of 0.7. This metric can be further improved by using the measurement-to-patient transition [17], which yields a value of 0.79 for patients.

The scattering signal from breast tissue collagen is not currently discernible using our bespoke laboratory diffractometers. However, the alterations in the collagen structure are correlated with another WAXS feature: the signal from the aqueous component. Despite the seeming simplicity of a single H_2_O molecule, water demonstrates a rich variety of structure-related properties [26,27]. Its interaction with light, primarily in the infrared range, led to the field of research known as Aquaphotomics [28]. In X-ray diffraction experiments, liquid water produces a broad maximum at ~*q* = 20 nm^−1^ that corresponds to the first-shell O–O correlations [29,30,31]. In biological tissues, the precise interaction of water with other components is critical and prominent. In particular, hydration layer water bridges play an essential role in the formation and characteristics of collagen fibrils [32,33,34]. Correspondingly, the X-ray diffraction signal from water is affected by the collagen structure (and vice versa), and its alternations can be observed in the water-related region of the diffraction patterns. The cancer/fibroadenoma classification is even better for the water region, with ROC AUCs for measurements and patients being 0.78 and 0.86, respectively.

A component of established models of invasive tumors is their association with preferentially aligned collagen, which promotes the motility of cancer cells. Therefore, given the structural intimacy of collagen and water, we hypothesize that this preferred orientation would also manifest within water signatures. Further, the collagen of benign lesions may not have this alignment characteristic, and, hence, discrimination may be possible using this feature. We show here that machine learning analysis of the water contributions to two-dimensional X-ray diffraction patterns and their Fourier transforms enable us to distinguish signals from fibroadenoma and cancer, both in the synchrotron and laboratory experiments.

## 2. Results

### 2.1. Measurements at the Diamond Light Source Synchrotron

**Figure 1:**
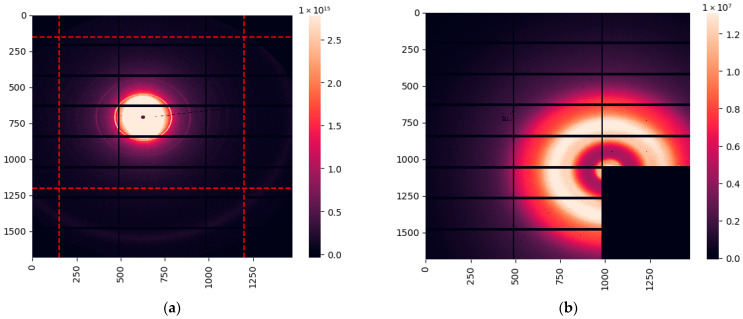

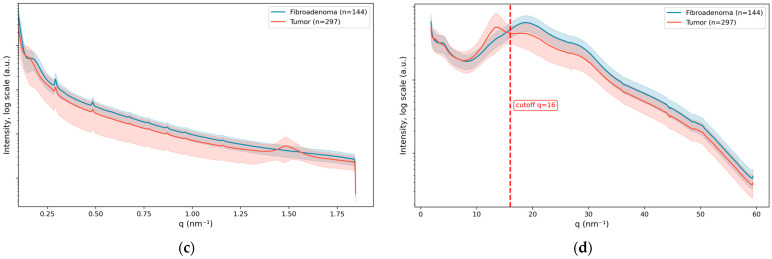
(**a**) SAXS image obtained at the Diamond Light Source synchrotron. Numbers indicate the position of the pixels on the detector. The red-dashed rectangle indicates the extracted boundary for collagen analysis. (**b**) WAXS image. The rectangular cutout in the bottom-right corner of the detector allows simultaneous measurement of the SAXS signal. (**c**) Azimuthally integrated SAXS profile. (**d**) Azimuthally integrated WAXS profile. The red dashed line indicates the cutoff for the analysis of water.

The SAXS and WAXS data were collected on beamline I22 at the Diamond Light Source [35], the UK’s national synchrotron facility. The samples from 24 cancerous patients and 12 patients with fibroadenoma were measured. In total, we obtained 297 SAXS and WAXS images of cancerous samples and 144 of fibroadenoma. Typical X-ray diffraction images are shown in Figure 1a for SAXS and in Figure 1b for WAXS. Figure 1c,d exhibit the dependencies of the intensity (averaged over all samples) on the momentum transfer, *q*, after azimuthal integration. For SAXS (Figure 1c), the amorphous scattering at very small angles is apparent, followed by several orders of the collagen reflexes and the triglyceride maximum at *q* = 1.5 nm^−1^. It is evident from this figure that the collagen peaks are similar between fibroadenoma and cancerous samples, whereas the triglyceride maximum is strongly suppressed in cancerous samples and completely absent in fibroadenoma. The cancer and fibroadenoma curves are almost coincident, making it impossible to classify based on azimuthally integrated features. For WAXS (Figure 1d), the situation is similar. The lipid maximum at 14 nm^−1^ is strongly suppressed in cancer and absent in fibroadenoma, and the broad water maxima are almost identical in the two cases.

### 2.2. Evaluation of the Collagen Signal at the Diamond Light Source Synchrotron

**Figure 2:**
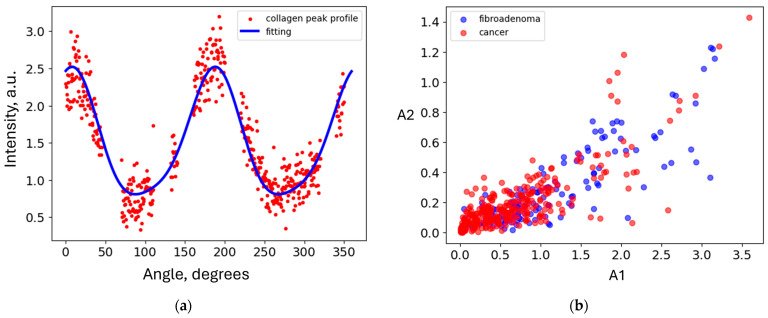
(**a**) The anisotropy of the signal at the 3rd order collagen peak at *q* = 0.3 nm^−1^. The blue line indicates the fitting based on two sinusoidal functions. (**b**) Measurements of cancerous (red) and fibroadenoma (blue) samples in the space of the two coefficients of the sinusoidal functions.

The azimuthal integration eliminates the anisotropy of the scattered signal, with the intensity depending on a single parameter: the distance to the center (or the momentum transfer *q*). However, the collagen scattering is anisotropic, as shown in Figure 2a. Here, we plotted the intensity of all the pixels belonging to the 3rd-order collagen peak at *q =* 0.3 nm^−1^ after correction of any anisotropy caused by the slightly non-circular shape of the incident beam. The correction protocol is described in Section 4.2.3. The least-squares fit of this data (the blue line) is given by

(1) $$I(\phi )=A_{0}+A_{1}\sin\left(2\phi +\phi_{1}\right)+A_{2}\sin\left(4\phi +\phi_{2}\right)$$

where *A*_0_ is a constant offset and *ϕ*_1_ and *ϕ*_2_ are the phase angles resulting from the random directions of the collagen fibrils. Each measurement can be represented by the two coefficients, *A*_1_ and *A*_2_, as demonstrated in Figure 2b. This figure illustrates that the cancerous and fibroadenoma clusters can be visibly separated. The logistic regression classification yields a sensitivity of 0.56 (95% CI: [0.5; 0.62]) and a specificity of 0.79 (95% CI: [0.73; 0.86]), where the numbers in the square brackets indicate the 95% confidence interval.

**Figure 3:**
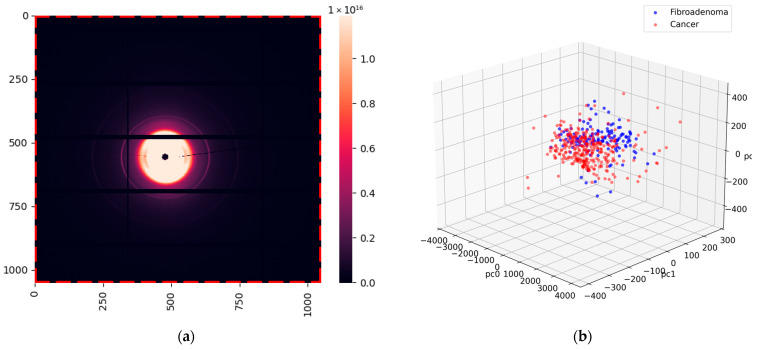

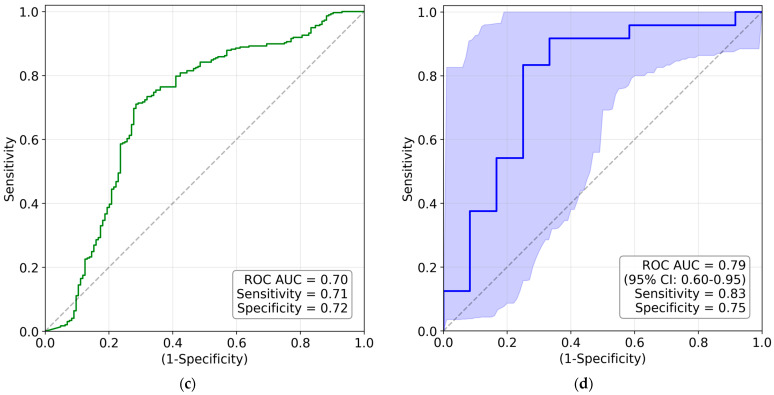
(**a**) SAXS image with excluded triglyceride peak; (**b**) the PCA-transformed data within the 3D PC space. Each PCA point corresponds to a single diffraction measurement; (**c**) measurement-based ROC curve for collagen contribution to synchrotron SAXS images; (**d**) patient-based ROC curve with the 95% confidence interval for collagen contribution to synchrotron SAXS images.

To further improve the classification metrics, we used the approach outlined in Section 4.2.3, which is based on two-dimensional Fourier transforms of the images and principal component analysis (PCA). To evaluate scattering in collagen only, we extracted the region within the red-dashed lines of Figure 1a. This allows us to eliminate the effect of the triglyceride peak at *q* = 1.5 nm^−1^ on classification. The resulting area is shown in Figure 3a. Each measurement is represented by a point in principal component space. The three-dimensional visualization is presented in Figure 3b. This figure shows that the cancer and fibroadenoma clusters are well separated, even in 3D. The ROC curve generated from 20 PC dimensions is demonstrated in Figure 3c. The AUC of 0.7 indicates significant separation of the clusters, with a sensitivity of 0.71 and a specificity of 0.72. However, these metrics can be further improved by implementing the measurement-to-patient approach developed in [17]. The corresponding ROC curve, with an AUC of 0.79 (95% CI: [0.6; 0.95]), is shown in Figure 3d. The patient-level sensitivity and specificity are 0.83 and 0.75. To ensure statistical significance, we performed a permutation test and, across 10,000 random label permutations, obtained a *p*-value of 0.0018, demonstrating the robustness of our results. As a complementary metric, we evaluated average precision (AP), i.e., the area under the precision–recall curve, and obtained a value of 0.86.

### 2.3. Evaluation of the Water Signal at the Diamond Light Source Synchrotron

**Figure 4:**
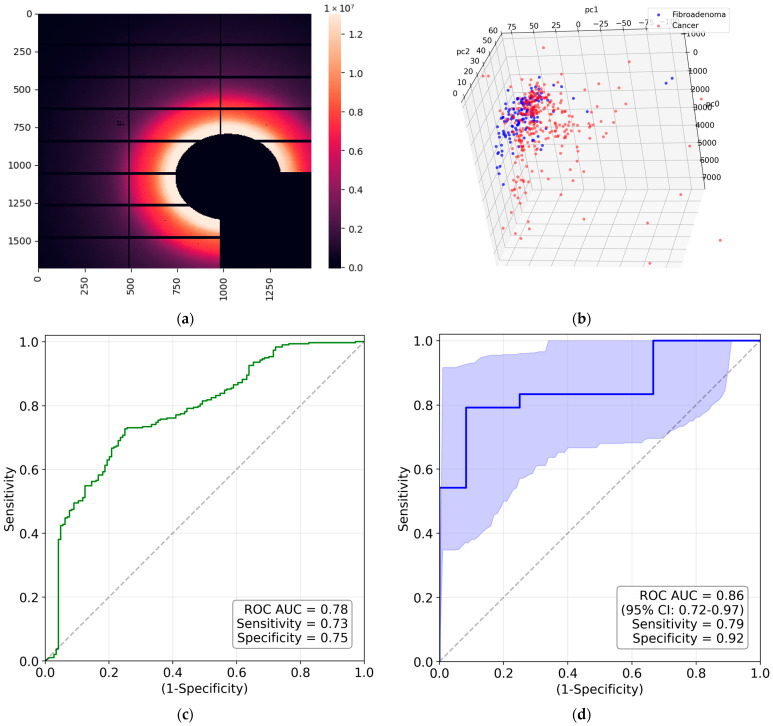
(**a**) WAXS image with excluded lipid maximum; (**b**) the PCA-transformed data within the 3D PC space. Each PCA point corresponds to a single diffraction measurement; (**c**) measurement-based ROC curve for water contribution to synchrotron WAXS images; (**d**) patient-based ROC curve with the 95% confidence interval for water contribution to synchrotron WAXS images.

For cancerous and fibroadenoma samples, the WAXS images obtained at the Diamond synchrotron are dominated by water-related maxima. The lipid contribution is less intense in these samples than in their healthy counterparts. However, to completely eliminate the lipid signal from our analysis, we extracted the region below 16 nm^−1^, as shown in Figure 1d. The resulting image for analysis is presented in Figure 4a.

To determine whether the water X-ray diffraction signal maintains the collagen-induced anisotropy, we evaluate the obtained diffraction pattern using the same approach as in the analysis of the collagen performed in the previous sub-section. Similarly, all measurements are shown as points in the 3D space of principal components in Figure 4b. Evidently, the cancer and fibroadenoma clusters are well-separated, with only a few outliers. The ROC curve is shown in Figure 4c, with an AUC of 0.78, a sensitivity of 0.73, and a specificity of 0.75. As in the case of collagen, these metrics improve with the measurement-to-patient transition, to an AUC of 0.86 (95% CI: [0.72; 0.97]), a sensitivity of 0.79, and a specificity of 0.92 (see Figure 4d). For these studies, the corresponding *p*-value is 0.0002, and the AP is 0.94, indicating a very robust classification with excellent metrics.

### 2.4. Measurements of Water Using the Laboratory Diffractometer

**Figure 5:**
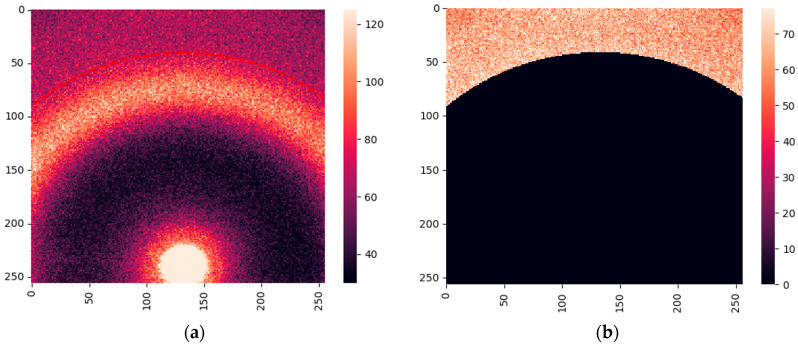
X-ray scattering images obtained at a laboratory diffractometer. Numbers indicate the position of the pixels on the detector. (**a**) Full image; (**b**) image with only water contribution. The red dashed line indicates the extracted boundary.

Measurements of the samples from the same 24 cancerous patients and 12 patients with fibroadenoma were performed on our bespoke laboratory diffractometer at Keele University. A typical WAXS pattern is shown in Figure 5a. Similar to the images obtained at the synchrotron, we extracted the regions with lipid responses below 16 nm^−1^, leaving only the contributions from water. The resulting image is presented in Figure 5b.

**Figure 6:**
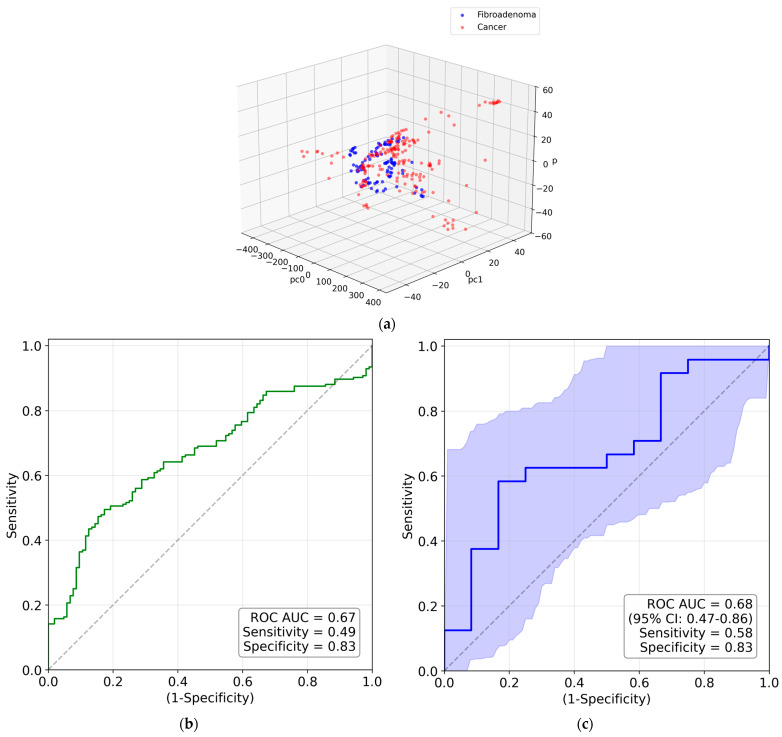
(**a**) The PCA-transformed data within the 3D PC space; (**b**) measurement-based ROC curve for water contribution to laboratory WAXS images; (**c**) patient-based ROC curve with the 95% confidence interval for water contribution to laboratory WAXS images.

The 3D PC space is shown in Figure 6a, with a visible separation between the cancerous and fibroadenoma clusters. The corresponding ROC curve is presented in Figure 6b. The metrics are poorer than for the synchrotron measurements, with an AUC of 0.73, a sensitivity of 0.80, and a specificity of 0.57. The patient-based metrics in Figure 6c are slightly improved. We obtained a significant *p*-value of 0.046 and an excellent AP of 0.86.

## 3. Discussion

In this study, we explored features of the X-ray scattering patterns of cancerous and fibroadenoma breast tissue samples. The previous comparison of cancerous and benign samples [17] was based on fat-molecule scattering. However, this was not effective for fibroadenoma, as its lipid signal was very similar to that of cancer. Here, we used different momentum-transfer ranges associated with collagen fibrils and water. The SAXS region was examined on a synchrotron, the Diamond Light Source, and the measurements of the WAXS region were performed both on the synchrotron and the laboratory diffractometer.

To explore the anisotropy of X-ray scattering from collagen, we did not use the standard azimuthal-integration approach, which has been shown to be ineffective for classification due to mitigation against orientation effects. Instead, we utilized a recently developed method of 2D Fourier transformations applied to tissue X-ray scatter [17,24,25]. Unlike classical equatorial/meridional analysis, the Fourier-based approach captures the presence and strength of anisotropy without explicit alignment, as directional order is encoded in the spatial-frequency magnitude distribution. We performed principal component analysis of the Fourier coefficients and classified the X-ray scattering images using logistic regression in a 20-dimensional PC space.

We obtained good classification metrics, which improved further when we classified patients rather than measurements, following the procedure described in [17]. The patient-based metrics were an ROC AUC of 0.79 (95% CI: [0.6; 0.95]), sensitivity of 0.83, specificity of 0.75, and AP of 0.86, with a *p*-value of 0.0018. We used logistic regression as a simple, robust classifier in our study to avoid overfitting with our limited dataset. The Random Forest classification for the same dataset yielded ROC AUC of 0.71 (95% CI: [0.51; 0.87]), sensitivity of 0.83, specificity of 0.58, and AP of 0.85, with a *p*-value of 0.024. The metrics obtained were inferior but still reasonable and should be improved in future studies with an increasing number of patients.

However, an excellent classification based on collagen molecules cannot be used in the clinical setting, as the resolution of collagen diffraction peaks is currently achievable only in synchrotron experiments or using impractical laboratory methods. We hypothesized that the anisotropy of collagen fibrils can manifest itself in the X-ray scattering signal from water. Water plays a vital role in the coupling of long collagen molecules, as various types of water bridges and hydration layers are formed. The periodicities of these bridge types are similar, being about 0.3 nm [32]. Correspondingly, we can expect the X-ray scattering signal to exhibit collagen-induced anisotropy at about 21 nm^−1^, i.e., near the usual water-scattering maximum associated with the tetrahedral structure.

A similar analysis using 2D Fourier transformation, PCA, and logistic regression in the 20-dimensional PC space provided an excellent cluster separation. The patient-based metrics were ROC AUC of 0.86 (95% CI: [0.72; 0.97]), sensitivity of 0.79, specificity of 0.92, and AP of 0.94, with the *p*-value of 0.0002, i.e., the classification was significantly improved and more robust than for the collagen itself. We attribute this to the anisotropic SAXS signal from the collagen being observed on a background of strong isotropic amorphous scattering. In contrast, water is the only scatterer in the WAXS region, with the momentum transfer exceeding 16 nm^−1^, i.e., greater than the lipid-related maximum. The corresponding Random Forest metrics were ROC AUC of 0.77 (95% CI: [0.60; 0.91]), sensitivity of 0.54, specificity of 1, and AP of 0.9, with the *p*-value of 0.0031.

The WAXS measurements were also performed using our laboratory diffractometer. As expected, the resulting metrics for the cancer/fibroadenoma classification were inferior to those obtained with the synchrotron but remained reasonable. However, we hope to achieve better results in future studies through straightforward improvements. In the current experimental arrangement, the exclusively water-related signal is measured over an observed azimuthal range much less than 180 degrees (see Figure 5b). This would be significantly extended in diffractometers with a larger detector active area. Such studies using a more balanced dataset and more patients in general are planned for the near future. We believe that the current work demonstrates the proof of principle for our approach to achieve cancer/fibroadenoma classification based on X-ray scattering from water with collagen-induced anisotropy.

## 4. Materials and Methods

### 4.1. Experimental Design

#### 4.1.1. Breast Tissue Specimens

All of the consented tissue samples (cancer and fibroadenoma) employed within this study were supplied by the Breast Cancer Now Biobank (BCNB) with National Research Ethics Service approval (23/EE/0229). Local ethical approval for the project was also obtained through the Keele University ethics committee (NS-210096). All of the specimens were fresh-frozen (FF) with approximate dimensions of 10 × 2 × 2 mm in size, taken from patients who consented to the BCNB, and all cases were supplied with a histopathological diagnosis acquired from conventional H&E sections. Each tissue specimen was initially placed and subsequently stored in a sample well (5 mm diameter, 2 mm depth) of a bespoke aluminum sample holder. The well was sealed with a SPEXTM 6 µm-thick mylar film to secure the tissue and mitigate against dehydration.

#### 4.1.2. X-Ray Diffraction (XRD) Measurements (Synchrotron)

Wide-angle X-ray scattering (WAXS) and small-angle X-ray scattering (SAXS) data were collected on beamline I22 at the Diamond Light Source (DLS) [35], the UK’s national synchrotron facility. The experiments were conducted using monochromatic X-rays with a wavelength of 0.1 nm, selected to optimize scattering contrast. A rectangular beam was collimated to 240 × 60 μm and employed for all data collections, as this provided appropriate spatial resolution for mapping structural heterogeneity within each specimen. Data acquisition was carried out using a 2D Pilatus P3-2M detector, Dectris AG, Baden-Daettwil, Switzerland, placed at a calibrated sample-to-detector distance of 170 mm for WAXS and 5935 mm for SAXS.

Each specimen, within its bespoke holder, was embedded in a custom-designed 9 × 9 aperture aluminum gel-solution sample grid. This grid was engineered to provide mechanical stability, uniform thermal conditions, and precise alignment during measurements. Further, this enabled batch processing of specimens.

For systematic data collection, a 5 × 5 Cartesian raster grid of measurement points was established for each specimen, covering the areas of interest. Based on preliminary optimization, the raster point spacing was 500 µm, a compromise between spatial resolution and data-acquisition efficiency. Data were recorded as fly-scans with 10 data frames per point to improve statistical reliability and reduce random noise. The acquisition time was fixed at 100 ms per frame.

The experimental conditions, including beam energy, sample environment, and data acquisition parameters, were rigorously controlled and documented to ensure reproducibility. All raw data were corrected, reduced, and subsequently processed using standard DLS pipelines, with detailed calibration steps and correction algorithms applied to account for detector geometry, background scattering, and beam intensity variations. This comprehensive methodology facilitates the reproducibility of our results and supports future comparative studies.

#### 4.1.3. X-Ray Diffraction (XRD) Measurements (Laboratory Diffractometer)

Laboratory XRD measurements were conducted using a bespoke X-ray diffractometer engineered and built by EosDx, Inc. (Menlo Park, CA, USA), a US-based company developing X-ray scattering for medical diagnostics. X-ray radiation from a copper target Incoatec Microfocus Source (Geesthacht, Germany) was harvested using two multilayer curved-mirror optics, yielding a monochromatic beam of 200 μm diameter and λ = 0.154 nm. A MiniPix SN1442 (ADVACAM, Prague, Czech Republic) two-dimensional detector was employed to detect and record the X-ray scatter. This detector has a square pixel size of 55 μm and an active array of 256 × 256 pixels. To calibrate the sample-to-detector distance (nominally 20 mm) and provide a quality assurance check, silver behenate powder (Thermo Scientific^®^ 045494.06, Heysham, Lancashire, UK) within an aluminium holder was scanned. For WAXS measurements, the measured mean *q*-position was 3.2271 nm^−1^ (expected: 3.2288 nm^−1^), yielding the standard deviation across patterns of ±0.0106 nm^−1^ and relative uncertainty of 0.33%. All experimental data were stored as a 256-by-256 matrix of integers representing total photon counts. Standard temperatures and pressures were maintained throughout all data collection. As in the synchrotron specimen, heterogeneity was assessed by acquiring data from several individual spots on each specimen (4–9, depending on sample size). Each individual scatter pattern was acquired over the *q*-range up to 23 nm^−1^ and collected for 30 s.

### 4.2. Data Analysis

#### 4.2.1. Image Preprocessing (Synchrotron)

A total of 297 WAXS and SAXS 2D scattering patterns corresponding to 12 fibroadenoma and 24 cancer patients were obtained at the DLS. Initial image processing was conducted using the standard beamline I22 pipelines [35] (systematic, pre-defined protocol steps for data processing) in conjunction with the DAWN data analysis software (Version 2.36.0) [36,37], designed to ensure the accuracy and reproducibility of the datasets.

A brief description of the pipeline process follows. Initially, the detector is calibrated to correct for inherent geometric aberrations and ensure accurate mapping. The scattering from standard NIST reference materials was measured to ensure accurate detector alignment and pixel-scale precision. A mask is generated for the detector active area that, when applied to the measured data, efficiently excludes artifacts such as the beam stop shadow and non-responsive (dead), or other inappropriately responding pixels. Edge effects are also removed by dilating the mask. The legitimacy of error estimation in photon-counting statistics was subsequently assessed using a Poisson model to validate the error calculations for measured intensities. For each measurement, beam-stop diode exposure measurements were used for channel averaging to reduce random noise. Further, this measurement provided correction data for variations in incident beam intensity. Each data collection was also corrected for any slight variations in exposure time, thus ensuring direct intensity comparisons. The next step was to average the intensities of the multiple frames collected at each measurement position, producing a single, high-fidelity 2D diffraction pattern for each measurement point. This stage also included background subtraction to isolate sample-specific scattering signals from inherent environmental and/or instrumental signals. Subsequently, inherent geometric distortion and absorption corrections were applied to each measurement point frame. As a final pipeline step, all non-numeric (NaN) values were removed, and, where necessary, intensities were scaled to standardize the datasets. This step ensured that the processed data were suitable for subsequent quantitative analyses.

Following this systematic processing pipeline, each measured position was associated with a well-calibrated and background-corrected diffraction profile suitable for quantitative structural analysis and comparative studies. The comprehensive documentation of each processing step enhanced data reproducibility and facilitated the validation of analytical results across independent data collections.

#### 4.2.2. Image Preprocessing (Lab Diffractometer)

A total of 288 WAXS images (frames) were obtained using the laboratory diffractometer from the same 12 fibroadenoma and 24 cancer patients. The unprocessed XRD data files contained intensities for each of the 256 × 256 pixels of the active detector array. Data processing and corrections followed closely those described in the DLS pipeline (Section 4.2.1). To eliminate the unwanted *q*-ranges, a mask was introduced (see Figure 3a, Figure 4a and Figure 5b), with the intensities of the pixels under the mask set to 0.

#### 4.2.3. Analysis of Anisotropy

The anisotropy of the observed scattered signal can be caused both by a natural anisotropy of the scatterers and by the non-circular shape of the photon source. To eliminate the latter from the collagen peak, we averaged the intensities of the two pixels at the same angle, shifted by 5 pixels in or out of the image center, and extracted this value from the intensity at the given pixel. The natural character of the remaining anisotropy is evident in the random phases 1 and 2 produced by the angle between the collagen fibril directions and the incident beam, which differs in each image.

#### 4.2.4. Fourier Coefficients Representation

In this work, we used the 2D Fourier coefficients approach proposed in [17,24,25]. Fourier coefficients were calculated for the preprocessed XRD data using the two-dimensional Discrete Fourier Transformation (2DFT) functions provided by the SciPy and NumPy libraries. This approach enabled the implementation of preprocessing analysis of the same area within each 2D image, thereby enhancing the influence of cancer on the XRD pattern and reducing the impact of any optical misalignment. The 2DFT application produced matrices of Fourier coefficients for each scatter frame. For all subsequent analyses, only the magnitudes of the complex Fourier coefficients were used. The elements of the acquired matrices were normalized by the element at (0,0), i.e., brightness. The spectrum was then centered (using the fftshift function) and converted to a log-magnitude map to compress the dynamic range and stabilize the variance.

In a procedure similar to that of Face Recognition [38,39], the Fourier coefficient matrices were ‘flattened’, i.e., translated into a single row. Subsequently, a single matrix was built by stacking the rows of all frames into a matrix, with the number of rows equal to the number of measured samples and the number of columns equal to the number of Fourier coefficients. This matrix was then used as the primary input to a principal component analysis (PCA). Accordingly, each sample was represented as a point in the PC space.

#### 4.2.5. Classification Procedure

To split the whole dataset into training and test subsets and perform cross-validation, we used GroupKFold/StratifiedGroupKFold from the scikit-learn library [40] with 5 folds, so each fold used ~80% for training and ~20% for testing. To avoid leakage from repeated measurements of the same patient, cross-validation was patient-grouped, with patient ID as the grouping variable. In each fold, all spots from a given patient were kept strictly in either the training or the test split. Inside each training fold, we fitted a scikit-learn pipeline consisting of standardization (StandardScaler), dimensionality reduction (PCA, 20 components), and a probabilistic classifier (default: Class-Weighted Logistic Regression). The metrics remained almost unchanged for PCs larger than 10, so we used 20 to be on the conservative side. Out-of-fold predicted probabilities were obtained for each measurement and then aggregated at the patient level by averaging across that patient’s data spots; the patient label corresponded to the measurement label. For comparison, we used the Random Forest classifier from the scikit-learn library.

Performance is reported at the patient level using ROC AUC and AP, computed from the patient-level out-of-fold predictions (scikit-learn metrics). For a single operating point, we selected a threshold using Youden’s J on the patient-level ROC curve and reported the corresponding sensitivity and specificity.

Uncertainty was quantified with a patient-level bootstrap (2000 resamples): we resampled N patients with replacement, kept their out-of-fold predicted probabilities and labels, recomputed ROC AUC and AP, and took the 2.5th–97.5th percentiles as the 95% confidence intervals. For the ROC-curve uncertainty band, an ROC curve was recomputed for each bootstrap resample, the true positive rate was interpolated onto a fixed false positive rate grid, and pointwise percentiles were used to form a 95% envelope. Finally, we assessed statistical significance using a patient-level permutation test on the same out-of-fold predictions, with the predicted scores held fixed. At the same time, patient labels were randomly shuffled (10,000 permutations) to generate a null distribution for AUC/AP under chance performance.

## 5. Conclusions

In conclusion, the present study shows that X-ray diffraction of collagen molecules and collagen-structured water can distinguish cancerous biopsy samples from fibroadenoma. This can complement the previously observed classification of cancerous and benign (excluding fibroadenoma) samples based on lipid reflexes and provide a rapid, accurate, and cost-effective triage procedure for histopathology.

## Data Availability

The files with the XRD patterns with extracted boundaries used for the analysis in this paper and the notebook with codes are available at https://doi.org/10.5281/zenodo.18421777. The codes are available upon request.
